# Effectiveness and safety of ear acupuncture for allergic rhinitis: A protocol of randomized controlled trial

**DOI:** 10.1097/MD.0000000000024943

**Published:** 2021-03-26

**Authors:** Xiaojun Ding, Shufen Huang, Yajun Tang, Jia Lin

**Affiliations:** a Ningbo Yinzhou District Baizhang Dongjiao Street Community Health Service Center; b Ningbo Yinzhou District Baihe Street Community Health Service Center; c Ningbo Hospital of Traditional Chinese Medicine, Ningbo, Zhejiang Province, China.

**Keywords:** allergic rhinitis, ear-acupressure, protocol, randomized controlled trial

## Abstract

**Background:**

Allergic rhinitis is a global problem. About 10% to 40% of the global population is affected by allergic rhinitis and is on the rise, which has a significant health and economic impact on society. Ear acupuncture is a non-invasive acupuncture therapy, which has been used in the treatment of allergic rhinitis, and some positive results have been reported, but there is not enough evidence to prove its efficacy and safety.

**Methods:**

This is a single-center, randomized, single-blind, sham-controlled trial. With the approval of the ethics committee of our hospital, participants with allergic rhinitis will be randomly assigned to receive either real or sham ear acupuncture once a week for 8 weeks, followed by 12 weeks of follow-up. Evaluate the patient's nasal symptom score and Standardised Rhinoconjunctivitis Quality of Life Questionnaire score, and monitor adverse events. Finally, the data are analyzed by SPSS 22.0 software.

**Discussion:**

The results of this study will determine the efficacy and safety of ear acupuncture in the treatment of allergic rhinitis and provide a basis for promoting the application of ear acupuncture in the treatment of allergic rhinitis.

**Trial registration:**

OSF Registration number: DOI 10.17605/OSF.IO/MVEF7.

## 1 Introduction

Allergic rhinitis (AR), also known as nasal allergy, is a non-infectious inflammatory disease of the nasal mucosa. It occurs after exposure to allergens, is mediated by IgE antibody and involves various immune cells and cytokines. Allergic rhinitis is a global problem, with the prevalence rate for adults and children being 14% and 13%, respectively, in the United States,^[1]^ It affects about 10% to 40% of the global population.^[2]^ It is known to interfere with sleep, attention, memory, quality of life, work, study,^[2–4]^ and driving ability posing a threat to traffic safety.^[5]^ In addition, AR is believed to be a risk factor for asthma, sinusitis, and other comorbidities.^[2,6]^ Current treatments for allergic rhinitis include minimizing exposure to allergens, the use of drugs to alleviate symptoms, anti-inflammatory therapy, and allergy immunotherapy (AIT).^[3]^ However, in clinical practice, it is difficult for many patients to control their symptoms effectively, whether they are treated with a single drug or a multi-drug combination of drugs,^[7]^ at the same time, there are some side effects,^[8]^ so there is an urgent need to find new alternatives.

In China, acupuncture is widely used in various diseases. It has the functions of regulating immunity and anti-inflammation through specific acupuncture point stimulation,^[9]^ and its efficacy has also been recognized by medical science in the world, becoming a new drug replacement therapy.^[10]^ Ear acupuncture (EAP) is a noninvasive semi–self-administered form of acupuncture. As a kind of acupuncture, auricular points have a long history in China. By stimulating specific acupoints on the ear in a non-invasive way, it has the functions of regulating endocrine, improving immunity and anti-allergy and so on. It is widely used in insomnia, obesity, acute and chronic pain, and other diseases.^[11–15]^ Previous studies have shown that EAP is also effective and safe for AR.^[16]^ But there is not enough evidence to prove this. Therefore, we plan to conduct a randomized, controlled, prospective clinical study to observe the efficacy and safety of EAP in the treatment of allergic rhinitis, so as to provide a basis for promoting the application of EAP in the treatment of allergic rhinitis.

## 2 Method

### 2.1 Study design

**Figure 1:**
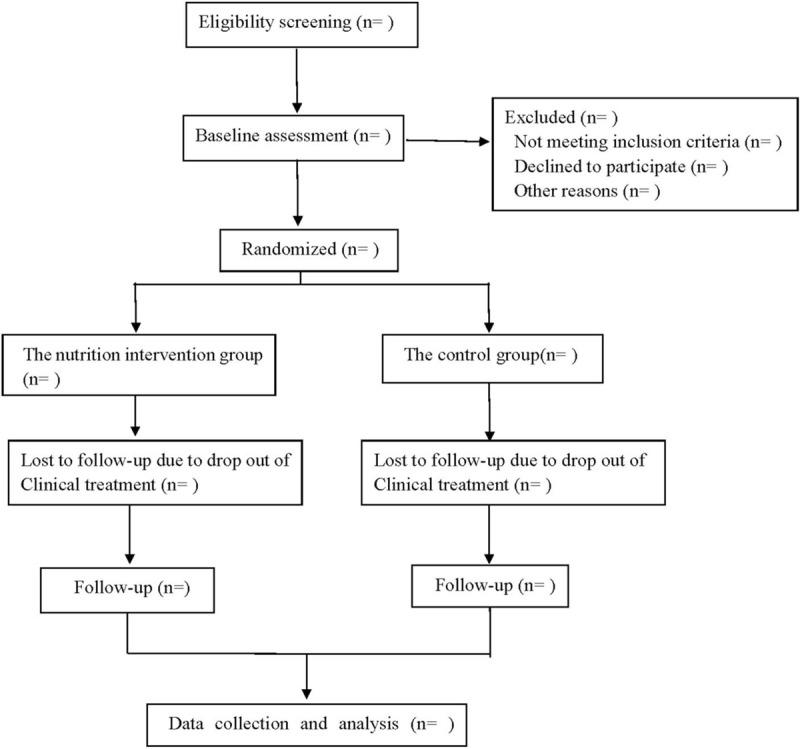
Flow diagram.

This study is a single-center, single-blind, randomized, and controlled clinical study. This study protocol has been approved by the Ethics Committee of our hospital and fully complies with the Helsinki Declaration in the process of implementation. The design, implementation, and results report of this scheme will be carried out in accordance with the requirements of SPIRIT 2013 Statement^[17]^ and CONSORT 2010 Statement.^[18]^ This research program has been registered in the open science framework (OSF) (registration number: DOI 10.17605/OSF.IO/MVEF7). The study flow chart is shown in Figure 1.

### 2.2 Patient population

#### 2.2.1 Participants source and sample size estimation

We will include patients through pre-hospital recruitment and screening of inpatients. After qualification assessment, patients will be informed of the protocol and purpose of the trial, and will be included in the study only after consenting to the study and signing an informed consent form.

Sample size estimation will be based on post-treatment Total nasal symptom score.^[19]^ According to the pre-experiment results, the real EAP group was 2.82 ± 1.48, and the Sham EAP group was 3.47 ± 1.57, α = 0.05, β = 0.2, and 107 samples were needed in each group according to the calculation by PASS15.0 software. Consider a drop-out rate of no more than 20%, with 134 cases in each group.

#### 2.2.2 Inclusion criteria

The patient was diagnosed with allergic rhinitis, and the diagnostic criteria refer to the 2008 WHO diagnostic description of allergic rhinitis,^[8]^ aged from 18 to 70 years, had at least 2 years history of typical allergic rhinitis symptoms, and had a positive reaction to any common allergen of allergic rhinitis.

#### 2.2.3 Exclusion criteria

1. Is undergoing systemic sex hormone therapy;
2. Combined with other active respiratory diseases, such as asthma, structural defects of the upper respiratory tract, etc.
3. Wearing hearing AIDS, unable to cooperate with the treatment;
4. EAP was used to treat other respiratory diseases in the past 6 months;
5. Allergic to therapeutic devices (e.g., tape, steel balls).

### 2.3 Randomization and blinding

In accordance with the principle of random distribution, the patients were randomly divided into treatment group (Real EAP group) and control group (Sham EAP group) by using computer-generated random number table. The designers and acupuncturists of this study are aware of the grouping due to the limitation of treatment. Participants and subjects involved in recruitment, evaluation, data entry, and data analysis were not informed of the grouping.

### 2.4 Intervention method

During the 8-week treatment period, participants received Real EAP or Sham EAP treatment once a week, and all treatments will be performed by the same registered acupuncturist (>5 years of treatment experience) to ensure participant-blinded and treatment consistency. Before the first treatment, the acupuncturist will provide the patient with technical information about the EAP and advise the patient on precautions. Points will be swabbed with 70% isopropyl alcohol before attaching the stainless steel pellets (1.2 mm in diameter, TaiHe, China), participants were asked to gently press the ball three times a day (morning, noon, and evening) to promote acupoint stimulation. Press each pellets for about 10 s, or until the ear turns red or slightly sore. In the subsequent treatment, the acupuncturist removed the previous treatment pellets once a week, and repeated the same steps on the other ear. There will be no skin penetration.

Five acupoints will be used for real EAP; these include shenmen (TF_4_), internal nose (TG_4_), lung (CO_14_), wind stream (SF_1,2i_), and adrenal gland (TG_2p_). They are selected on the basis of traditional Chinese medicine theory, previous clinical research, and expert consultation.^[16,20]^ Five sham EAP acupoints are helix 2 (HX_10_), shoulder (SF_4,5_), clavicle (SF_6_), OCCIPUT (AT_3_), and tooth (LO_1_). These acupoints have no effect on the relief of allergies or any nasal symptoms.

### 2.5 Outcome measures

The main outcome indicator is the decrease of Total nasal symptom score^[19]^ (including self-assessment in several aspects of nasal congestion, runny nose, nasal itching, sneezing, difficult sleep). The secondary outcome indicators are Standardized Rhinoconjunctivitis Quality of Life Questionnaire (RQLQ)^[21]^ and mitigate changes in drug use. Data were recorded by Case Report Forms (CRFs) during treatment (8 weeks, weekly) and follow-up (12 weeks, every 3 weeks). Considering the possibility of pellets falling off during treatment, participants were asked to record how many pellets were still attached to their auricular points in the CRFs every day. The total number of pellets remaining each week was analyzed as treatment dose data.

### 2.6 Adverse events and termination of trial

For safety reasons, we will record all unexpected reactions related to the study treatment with detailed description, including time of occurrence, duration of symptoms, severity of symptoms, management measures, time when adverse reactions disappear and classification of causality. The investigator will document and manage all adverse events, whether or not they are related to the study treatment. If more than 25% of patients stop the intervention due to adverse events, the superior physician will make the decision to terminate the trial. The case report form (CRFs) will be used for data collection to record demographics, assessments, and reasons for patient withdrawal. At the end of the study, the investigators will submit the CRFs form to the data Management Committee.

### 2.7 Data collection and management

The data of this study were collected by two assistants and entered into a pre-designed table. The relevant information and data of this study will be collected, shared, and stored in a separate repository to protect the confidentiality before, during and after the test. People outside the research group have no access to the relevant information. Without the written permission of the participants, the research information of the participants will not be published outside the study.

### 2.8 Statistical analysis

The data were analyzed by SPSS.22 software package, the hypothesis test was two-tailed test, and the α level was set to *P* < .05. The measurement data in accordance with normal distribution and homogeneity of variance are tested by independent sample *T* test, while those inconformable are tested by Mann–Whitney *U* test.

### 2.9 Study monitoring

The hospital clinical trial management department will be responsible for monitoring the progress of this study, and the organization will not be involved in the study at all, and there will be no conflict of interest. On-site monitoring meetings are held before and after the start of the study and quarterly to monitor the progress of the study and to ensure that it is carried out in accordance with established protocols, good clinical practice guidelines and applicable regulatory requirements. Although a separate data monitoring committee will not be established, the hospital clinical trial management department will perform this function by regularly monitoring this clinical trial to ensure scientific validity, scientific integrity, and data accuracy.

## 3 Discussion

Allergic rhinitis is often induced by weather changes, pollen, animal hair, diet and other allergic factors, the main clinical manifestations are continuous episodes of paroxysmal sneezing, a large number of watery snot, nasal congestion and itching, and some patients have decreased sense of smell, affecting the quality of life.^[22]^ AR is an allergic reaction mediated by IgE under the chemotaxis of various inflammatory cytokines after specific individual contact with antigen. The nasal mucosa produces various pathological changes, and the excitability of the nasal parasympathetic nerve is too strong, resulting in vascular dilation, enhanced permeability, excessive secretion of serous cells,^[23]^ modern medicine is mainly based on drug treatment, Medications include oral and topical histamine H1 receptor antagonists, topical and systemic glucocorticosteroids, chromones, decongestants, topical anticholinergics, antileukotrienes, and oral anti-allergic drugs.^[24]^ However, the adverse reactions caused by it are still a common concern of doctors and patients, and the curative effect is limited, unable to completely relieve the symptoms^[25]^ Therefore, it is necessary to find alternative treatment options for patients with allergic rhinitis.

Ear acupuncture has the characteristics of simple operation, less pain, less side effects and lasting and stable effect. Clinical studies have proved that lung (CO_14_) and wind stream (SF_1,2i_) can relieve congestion and runny nose, adrenal gland (TG_2p_) can resist inflammation and allergy, and its mechanism of action may be to regulate the imbalance of Th1/Th2 cells by inhibiting the differentiation of Th cells to Th2, thereby reducing the synthesis of IgE and inhibiting the occurrence of allergic reactions.^[26]^ Although EAP has been widely used in the treatment of allergic rhinitis in China, there is still a lack of strict clinical studies to verify its curative effect. The existing clinical studies mostly use EAP combined with other treatment schemes, or compare the curative effect with western medicine, and many confounding factors interfere with our evaluation of its curative effect.^[27,28]^ Therefore, we designed this randomized controlled study to determine the efficacy of EAP in the treatment of allergic rhinitis by comparing with sham EAP.

In addition, there are still some deficiencies in the design of this study. First of all, due to the limited treatment methods, this study cannot carry out double-blind design, which may lead to certain bias. Second, this study is a single-center study, and the source of the case is relatively single and limited, which may affect the conclusions of the study to some extent.

## Author contributions

**Conceptualization:** Xiaojun Ding, Shufen Huang.

**Data curation:** Shufen Huang, Yajun Tang.

**Formal analysis:** Xiaojun Ding.

**Funding acquisition:** Xiaojun Ding.

**Software:** Yajun Tang, Jia Lin.

**Supervision:** Yajun Tang.

**Writing – original draft:** Xiaojun Ding, Shufen Huang.

**Writing – review & editing:** Xiaojun Ding.
